# Emergence of equine-like G3 strains as the dominant rotavirus among children under five with diarrhea in Sabah, Malaysia  during 2018–2019

**DOI:** 10.1371/journal.pone.0254784

**Published:** 2021-07-28

**Authors:** Lia Natasha Amit, Daisuke Mori, Jecelyn Leaslie John, Abraham Zefong Chin, Andau Konodan Mosiun, Mohammad Saffree Jeffree, Kamruddin Ahmed

**Affiliations:** 1 Faculty of Medicine and Health Sciences, Department of Pathobiology and Medical Diagnostics, Universiti Malaysia Sabah, Kota Kinabalu, Sabah, Malaysia; 2 Faculty of Medicine and Health Sciences, Borneo Medical and Health Research Centre, Universiti Malaysia Sabah, Kota Kinabalu, Sabah, Malaysia; 3 Faculty of Medicine and Health Sciences, Department of Community and Family Medicine, Universiti Malaysia Sabah, Kota Kinabalu, Sabah, Malaysia; 4 Kunak District Health Office, Ministry of Health Malaysia, Kunak, Sabah, Malaysia; University of Malaya, MALAYSIA

## Abstract

Rotavirus infection is a dilemma for developing countries, including Malaysia. Although commercial rotavirus vaccines are available, these are not included in Malaysia’s national immunization program. A scarcity of data about rotavirus genotype distribution could be partially to blame for this policy decision, because there are no data for rotavirus genotype distribution in Malaysia over the past 20 years. From January 2018 to March 2019, we conducted a study to elucidate the rotavirus burden and genotype distribution in the Kota Kinabalu and Kunak districts of the state of Sabah. Stool specimens were collected from children under 5 years of age, and rotavirus antigen in these samples was detected using commercially available kit. Electropherotypes were determined by polyacrylamide gel electrophoresis of genomic RNA. G and P genotypes were determined by RT-PCR using type specific primers. The nucleotide sequence of the amplicons was determined by Sanger sequencing and phylogenetic analysis was performed by neighbor-joining method. Rotavirus was identified in 43 (15.1%) children with watery diarrhea. The male:female ratio (1.9:1) of the rotavirus-infected children clearly showed that it affected predominantly boys, and children 12–23 months of age. The genotypes identified were G3P[8] (74% n = 31), followed by G1P[8] (14% n = 6), G12P[6](7% n = 3), G8P[8](3% n = 1), and GxP[8] (3% n = 1). The predominant rotavirus circulating among the children was the equine-like G3P[8] (59.5% n = 25) with a short electropherotype. Eleven electropherotypes were identified among 34 strains, indicating substantial diversity among the circulating strains. The circulating genotypes were also phylogenetically diverse and related to strains from several different countries. The antigenic epitopes present on VP7 and VP4 of Sabahan G3 and equine-like G3 differed considerably from that of the RotaTeq vaccine strain. Our results also indicate that considerable genetic exchange is occurring in Sabahan strains. Sabah is home to a number of different ethnic groups, some of which culturally are in close contact with animals, which might contribute to the evolution of diverse rotavirus strains. Sabah is also a popular tourist destination, and a large number of tourists from different countries possibly contributes to the diversity of circulating rotavirus genotypes. Considering all these factors which are contributing rotavirus genotype diversity, continuous surveillance of rotavirus strains is of utmost importance to monitor the pre- and post-vaccination efficacy of rotavirus vaccines in Sabah.

## Introduction

Rotavirus is a nonenveloped double-stranded RNA virus belonging to the family Reoviridae [1]. Its genome is composed of 11 gene segments that are enclosed by an inner core, an innermost layer that is interconnected with the outer capsid by the intermediate capsid. The outer capsid is composed of glycosylated VP7 protein and spiky protease-sensitive VP4 protein [2,3]. VP4 and VP7 proteins contain epitopes that induce neutralizing antibodies [4,5]. These proteins also define the P and G genotypes, which are combined to produce the dual rotavirus classification system. To date 36 G and 51 P genotypes have been identified in rotaviruses [6]. G1, G2, G3, G4, G9, P4, P6 and P8 genotypes are those commonly detected in circulating strains. Globally, G1P[8], G2[P4], G3P[8], G4P[8], G9P[8]/P[6] and G12P[8]/P[6] are the dominant genotypes detected during human infections [7,8]. Moreover, novel rotaviruses with distinct genotypes have been detected as a result of gene reassortment between human and animal rotaviruses [9,10].

Rotaviruses can also be classified based on the migration pattern of the segmented genome in polyacrylamide gel electrophoresis (PAGE), termed the electropherotype. There are two major patterns of electropherotypes, long and short, differentiated by the migration rate of segment 10 containing the NSP5 gene. This results from the insertion of AT-rich sequences in the 3-terminal noncoding region of segment 11 [11], which causes an inversion of the migration order of gene segments 10 and 11 [8]. There is a general correlation between G/P type and electropherotypes. G1P[8], G3P[8], G4P[8] and G9P[8] strains show a long electropherotype pattern, whereas G2P[4] shows a short electropherotype pattern.

Two commercially available vaccines, Rotarix and RotaTeq, have been recommended by the World Health Organization (WHO) for all national immunization programs and particularly for countries in south and southeastern Asia and sub-Saharan Africa [12], after they resulted in substantial reductions in severe rotavirus infections in industrialized countries [13,14]. In Malaysia, both rotavirus vaccines are available on the private market and as of July 15, 2019. Rotarix is a monovalent vaccine derived from a human G1P[8] isolate. RotaTeq is pentavalent, consisting of a mixture of human bovine rotavirus monoassortants, carrying the genes encoding the human G1, G2, G3, G4 and P[8] proteins on the genetic background of a bovine rotavirus G6P[5] [15]. As of April 2018, 95 countries have introduced rotavirus vaccines into their national immunization programs, including many low-income countries [16]. Other than these vaccines, several countries are developing their own rotavirus vaccines based on the predominant circulating genotypes in these respective countries; for example, in 2016, India introduced Rotavac, a monovalent vaccine derived from a G9P[11] strain [16,17]. This vaccine is listed as a prequalified vaccine by the WHO [16]. In 2018, India included Rotasil, a multivalent (G1, G2, G3, G4, G9 and P[8]) rotavirus vaccine, in its national immunization program [18].

Rotavirus remains the most significant causative agent of acute gastroenteritis among children under 5 years of age worldwide and has been implicated in an estimated 25 million hospitalizations and 450,000 deaths annually [19]. Developing countries in Asia and Africa have a high burden of rotavirus infection and account for over 90% of deaths, according to an estimate of 2015 [20]. In Malaysia, rotavirus is responsible for 31,000 hospitalizations, 41,000 outpatient visits, 145,000 episodes of home-treated gastroenteritis, and 27 deaths per year [21]. Studies in Malaysia of rotavirus circulating genotypes have been reported from the 1970s to the 2000s [22]. The common rotavirus genotypes, G1P[8], G2P[4], G3P[8] G9P[8], were identified and their predominance has changed from time to time [22]. However, starting from 2000, G9P[8] has emerged to become the predominant rotavirus identified in Johor (42%) [23], Kuala Lumpur, and Kuching (73.3%) [23,24]. Continuous surveillance of rotavirus genotypic distribution at a regional level is crucial to ensure that Rotarix and RotaTeq provide protection against the circulating strains. However, most of the studies on rotavirus have been done in west Malaysia, particularly in Kuala Lumpur [22–24].

Malaysia consist of 13 states and three federal territories, broadly divided into West and East Malaysia (S1 Fig). West Malaysia comprises the Malay Peninsula. East Malaysia consists of two states and one federal territory on Borneo island. However, rotavirus susceptibility varies in different areas; for example, children in East Malaysia are more vulnerable to diarrhea than those in West Malaysia (8.7 hospitalizations/1,000 children vs 4.3 hospitalizations/1,000 children). Furthermore, children from the indigenous community have a higher rate of hospitalization (11.6 hospitalizations/1,000 children) because of diarrhea than other ethnicities (2.9–8.0 hospitalizations/1,000 children) [25].

Except for one study done in Queen Elizabeth Hospital, Kota Kinabalu during 2005–2006 which found that 16% of samples positive for rotavirus infection [26], no studies have been conducted in the state of Sabah despite the importance of this virus and the availability of a vaccine. There have also been no published studies of the genotype distribution of rotaviruses in Malaysia over the past 20 years. Moreover, there have been no studies of the genotype distribution of rotavirus among children of Sabah, which is crucial before introducing a rotavirus vaccine. Therefore, the present study was conducted to determine the circulating genotypes among children under 5 years of age in Sabah, which could be useful for informed policy-making for rotavirus vaccine implementation in this state.

## Materials and methods

### Collection of watery stool samples and patients’ information

From January 2018 through March 2019, watery stool samples were collected from children under 5 years of age with diarrhea attending Sabah Women and Children’s Hospital, Kunak District Hospital, Menggatal Health Clinic and Telipok Health Clinic. Kunak District Hospital is situated in the Kunak district, and the other hospitals are in the Kota Kinabalu district. A case of diarrhea was defined as passing of three loose stools during a 24-hour period (27) and convenient sampling method was used to collect sample. Before sample collection written informed consent was taken from the guardians of the children. The age, gender and race of the children were recorded. The collected stool samples were sent in cold chain to the laboratory of Universiti Malaysia Sabah, Kota Kinabalu. The samples were stored in -80 C until used.

### Ethics approval

Ethics approval was obtained from the National Medical Research Registrar for the Telipok and Menggatal Health Clinics (NMRR-16-2245-32787), Sabah Women and Children’s Hospital (NMRR-19-3925-52370), and Kunak District Hospital (NMRR-20-1324-55178).

### Rotavirus identification

Stool samples were diluted tenfold in phosphate-buffered solution. Rotavirus was identified using a commercial enzyme immunoassay according to the manufacturer’s instruction (Rotaclone, Meridien Diagnostics Inc, Cincinnati, USA).

### RNA extraction and determination of G and P genotype of rotavirus

Rotavirus genomic RNA was extracted from rotavirus-positive samples using a QIAamp Viral RNA Mini Kit according to the manufacturer’s instructions (Qiagen GmbH, Hilden, Germany). The VP7 and VP4 genes were amplified by reverse transcription-polymerase chain reaction (RT-PCR) amplification [27] using AccessQuick RT PCR MasterMix (Promega Corporation, Madison, WI, USA). For G genotyping, primers VP7-R, G1, G2, G4, and G8 were used as described previously [28], while for G3 and G9 genotypes, the primers used were described by Gouvea et al., [28]. Primers for P genotyping were used according to Gunasena et al. [29]. Respective genotypes were determined primarily by estimating the molecular weight of the amplicons after running in agarose gel. Nucleotide sequencing of the amplicons were determined; genotypes were determined by submitting nucleotide sequences to the rotavirus genotyping tool (https://www.viprbrc.org/brc/rvaGenotyper.spg?method=ShowCleanInputPage&decorator=reo).

### Nucleotide sequencing, phylogenetic analyses and nucleotide identity

The nucleotide sequence of the amplicons was determined using the BigDye Terminator Cycle Sequencing Kit (v. 3.1; Applied Biosystems, Foster City, CA, USA), according to the manufacturer’s instructions, and the product was run on an ABI Prism 3100 Genetic Analyzer (Applied Biosystems). Nucleotide sequences of the VP7 and VP4 genes of Sabahan strains and other strains retrieved from GenBank were used for phylogenetic analyses. Phylogenetic analyses were done with the neighbour joining method using MEGA X (https://www.megasoftware.net) after aligning the nucleotide sequences using CLUSTAL W. The branching patterns were evaluated using a bootstrap analysis of 1,000 replicates. The nucleotide identities of the VP7 and VP4 genes of different lineages were calculated by online software (www.bioinformatics.org).

### Determination of electropherotypes

The genomic dsRNA was extracted from rotavirus-positive samples and electropherotype was determined by subjecting the extracted dsRNA to PAGE according to a previously published method [30,31]. In brief, 5 μl of extracted dsRNA mixed with 5 μl of loading buffer was loaded in each lane of a 10% polyacrylamide gel and run for 16 h at constant a current of 8 mA. Numbering of electropherotypes was arbitrarily assigned and based upon distinct changes in the migration patterns within at least one of the four groups of segments, i.e., segments 1 to 4, 5 and 6, 7 to 9, or 10 and 11.

## Results

**Fig 1:**
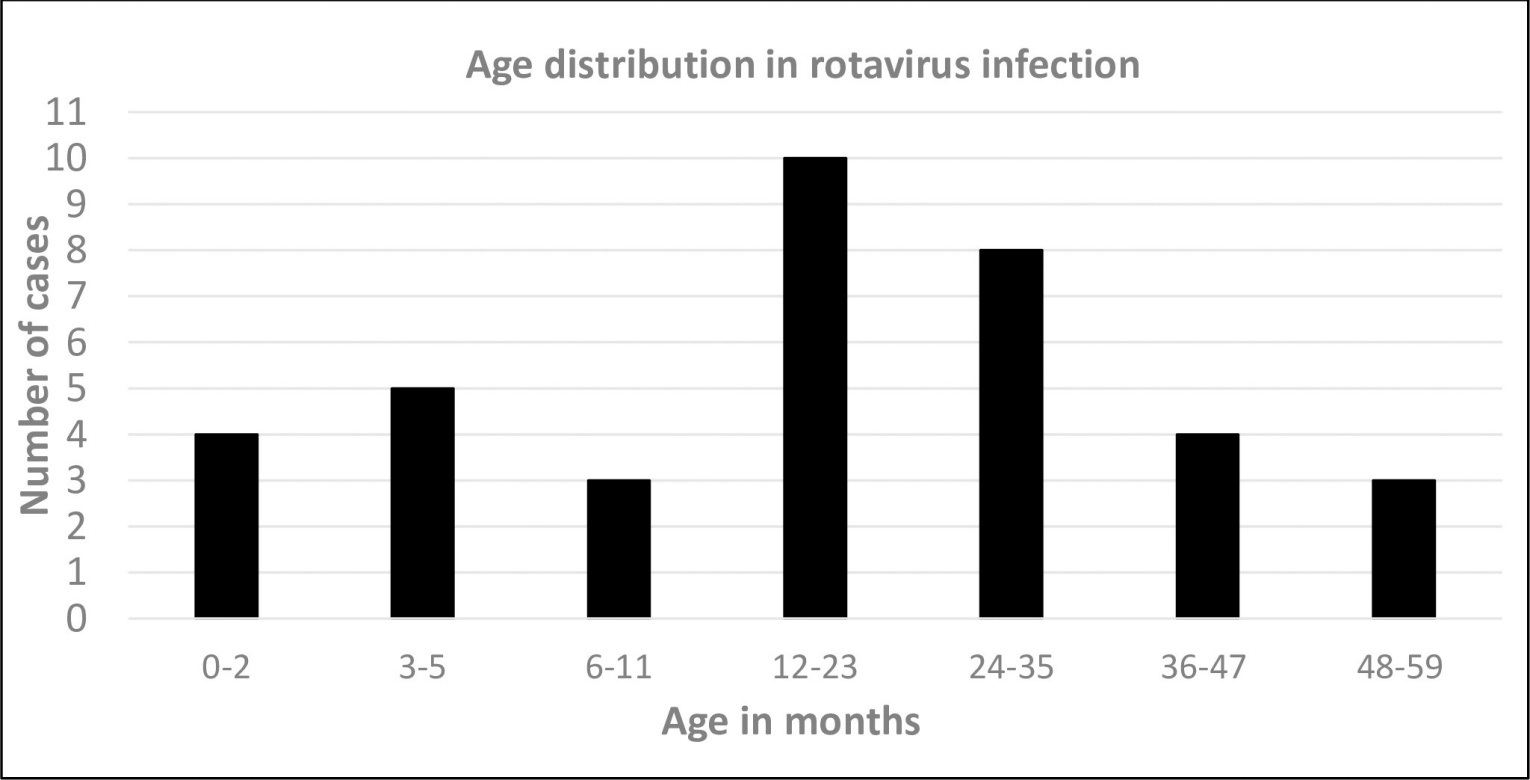
Age distribution. The number of cases with rotavirus diarrhea distributed according to age.

A total of 285 samples were collected during the study period. The male:female ratio of the subjects was 1.5:1 (153 male, 102 female and 30 undetermined). Their median age was 18 months and ranged from 15 days to 66 months (only one child was 66 months). Rotavirus was detected in 15.1% (43/285) of the samples. The male:female ratio of the rotavirus-infected children was 1.9:1. The median age of patients with rotavirus diarrhea was 14 months and ranged from 15 days to 55 months. Rotavirus was identified most commonly in children aged 12–23 months, followed by those aged 24–35 months (Fig 1). Rotavirus was less likely to affect children aged 6–11 months and 48–59 months.

Forty-two rotavirus-positive samples were available for G and P genotyping. The predominant G genotype was G3 (74% n = 31) followed by G1(14% n = 6), G12(7% n = 3), G8(2% n = 1), and in one sample (2%) the G genotype could not be determined. P genotypes were identified in 42 rotavirus-positive samples. The predominant P genotype was P[8], which was detected in 39 (93%) of the samples, followed by P[6] in 3 (7%) of the samples. Based on the G and P genotype combinations, the predominant genotype was G3P[8] (74% n = 31), followed by G1P[8] (14% n = 6), G12P[6](7% n = 3), G8P[8](3% n = 1), and GxP[8] (3% n = 1).

**Fig 2:**
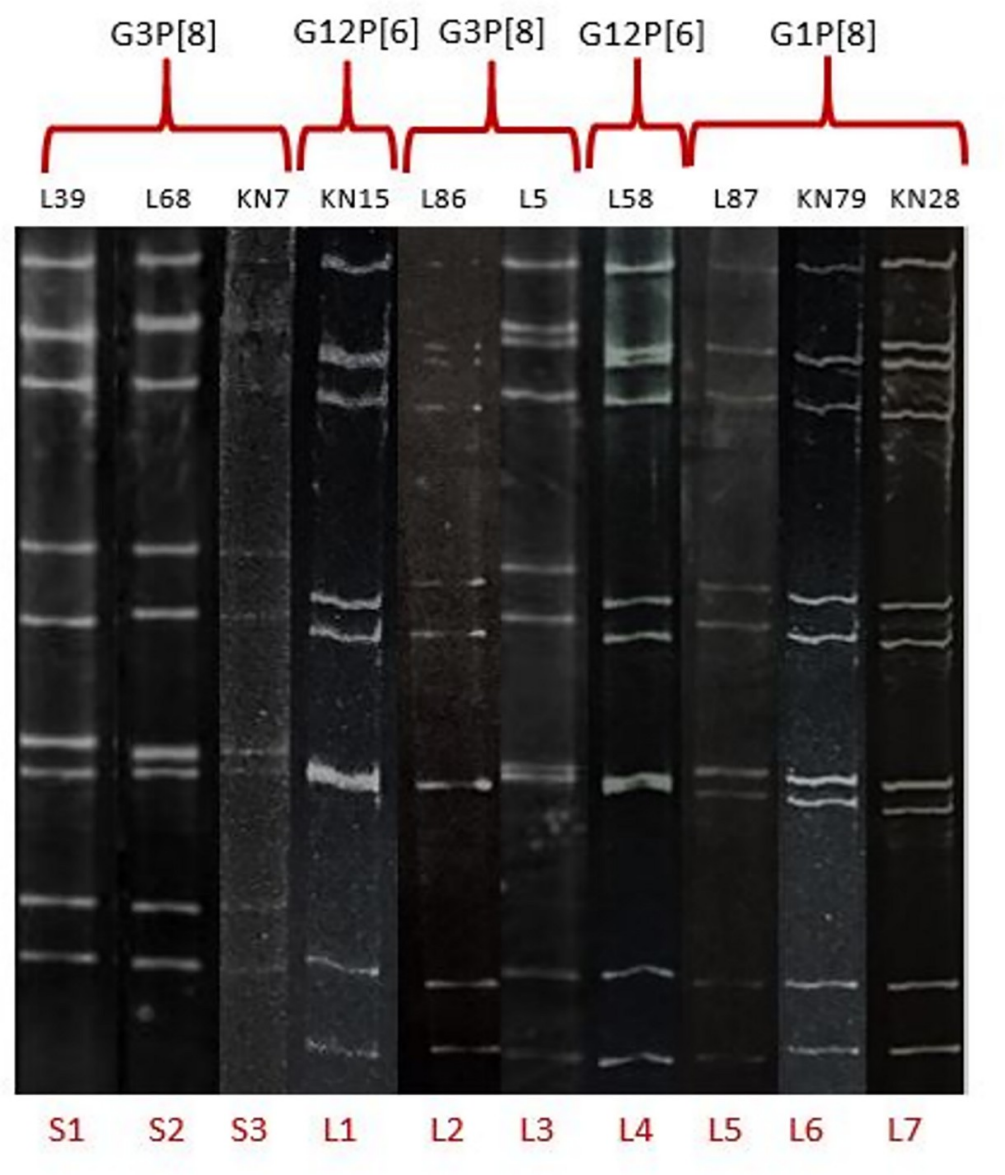
Electropherotypes of rotaviruses identified in Sabah. In total, 11 electropherotypes were identified, three short (S1–S3) and eight long (L1–L8) electropherotype patterns were identified. The strain number and genotype of each electropherotype is shown above the electropherotype.

Of the samples subjected to PAGE, all 11 segments could be identified in 34 samples and their electropherotypes could be determined. All 11 segments were not visible in remaining samples because genomic RNA yield was less possibly due to the lower concentration of virus particles. Three short and eight long electropherotype patterns were identified (Fig 2). Among the short electropherotypes, 11 strains belonged to S1, 11 to S2 and 1 to S3 electropherotype. Among the long electropherotypes, there were two each of L1, L2 and L3 electropherotypes. There was one strain of each electropherotype L4, L5, L6, L7 and L8.

**Fig 3:**
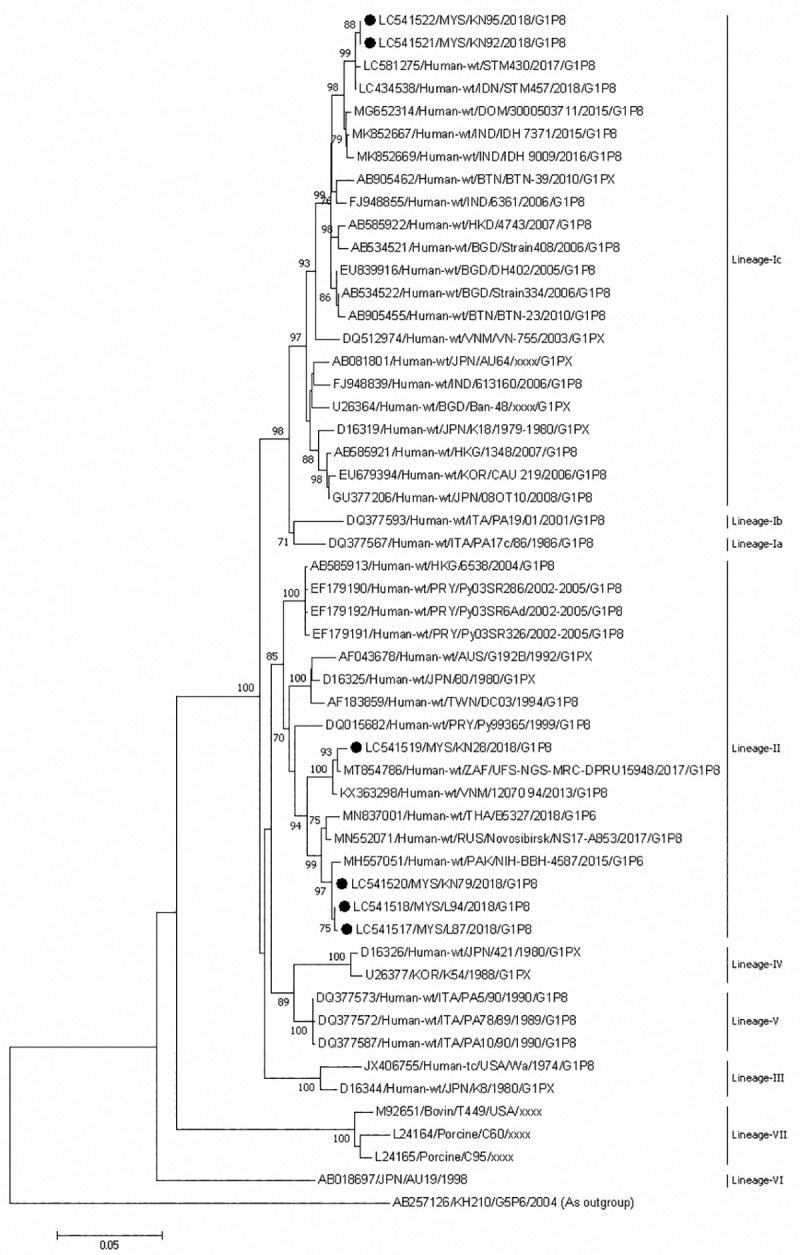
Phylogenetic tree constructed with the nucleotide sequences of the VP7 gene of G1 strains. Human rotavirus KH210 (G5) was used as an outgroup. The number adjacent to the node represents the bootstrap value and values lower than 70% have not been indicated. Scale bar shows genetic distance expressed as nucleotide substitutions per site. The strains identified in this study are marked with a filled circle. Strains from Sabah belong to lineage Ia, and II. The nucleotide sequences of our strains have been submitted to the databases of the DNA DataBank of Japan, the European Molecular Biology Laboratory, and GenBank. The accession numbers are shown at the beginning of each strain.

Phylogenetic analysis of the VP7 gene of genotype G1 showed that two G1 rotavirus strains from Sabah formed an independent cluster but were closely related to strains from Indonesia and belonged to lineage Ic (Fig 3). The VP7 genes Sabahan strains shared 99.9% nucleotide identity among themselves and 99.5–99.8% identity with Indonesian strains. One G1 strain formed a cluster with strains from South Africa and Vietnam, and belonged to lineage II. These strains shared 99.2–99.5% nucleotide identity among themselves. The other three strains from Sabah also belonged to lineage II and clustered with a strain from Pakistan. There was 99.8–99.9% nucleotide identity among strains from Sabah. Sabahan strains shared 99.5–99.6% nucleotide identity with Pakistani strain.

**Fig 4:**
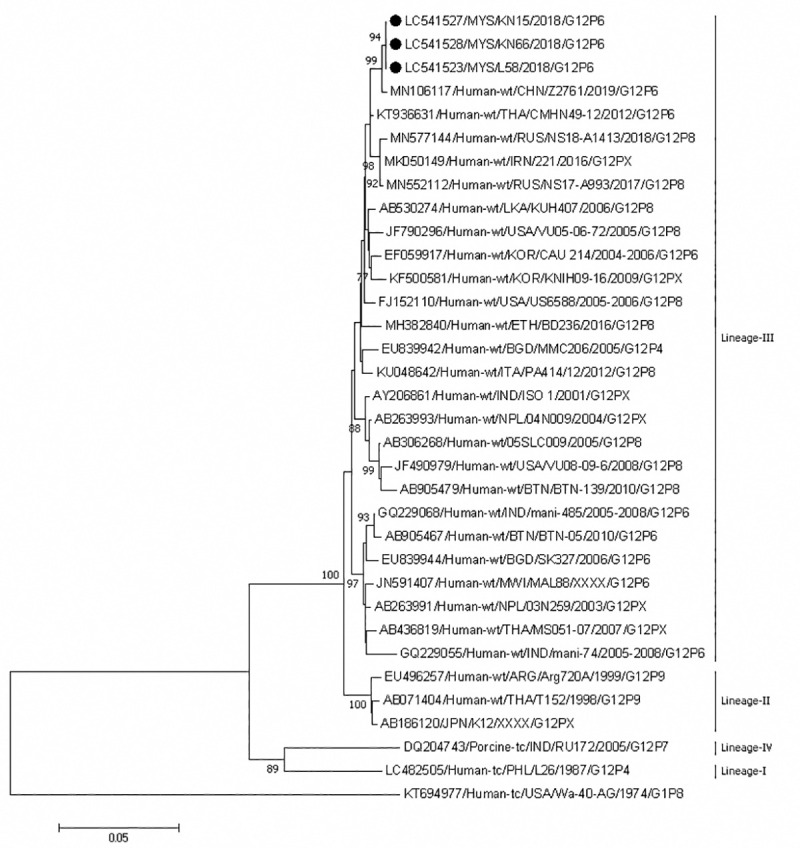
Phylogenetic tree constructed with the nucleotide sequences of the VP7 gene of G12 strains. Human rotavirus Wa-40-AG (G1) was used as an outgroup. The number adjacent to the node represents the bootstrap value and values lower than 70% have not been indicated. Scale bar shows genetic distance expressed as nucleotide substitutions per site. The strains identified in this study are marked with a filled circle. Strains from Sabah belong to lineage III. The nucleotide sequences of our strains have been submitted to the databases of the DNA DataBank of Japan, the European Molecular Biology Laboratory, and GenBank. The accession numbers are shown at the beginning of each strain.

**Fig 5:**
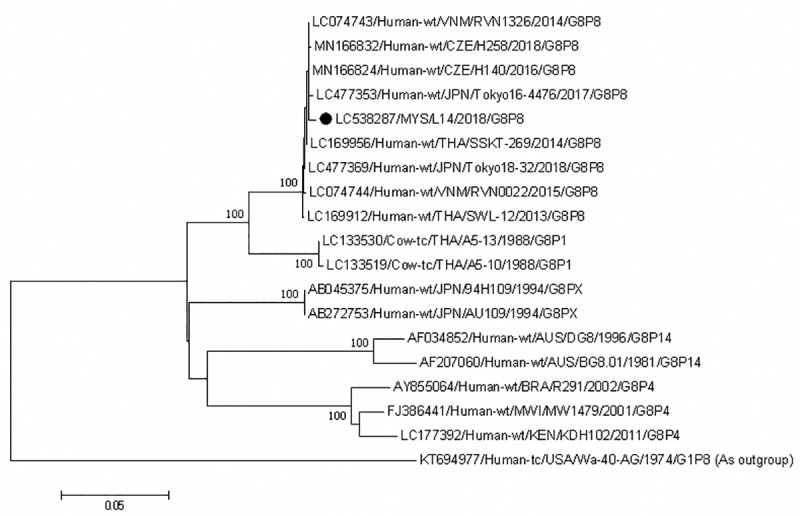
Phylogenetic tree constructed with the nucleotide sequences of the VP7 gene of G8 strains. Human rotavirus Wa-40-AG (G1) was used as an outgroup. The number adjacent to the node represents the bootstrap value and values lower than 70% have not been indicated. Scale bar shows genetic distance expressed as nucleotide substitutions per site. The strains identified in this study are marked with a filled circle. The nucleotide sequences of our strains have been submitted to the databases of the DNA DataBank of Japan, the European Molecular Biology Laboratory, and GenBank. The accession numbers are shown at the beginning of each strain.

Phylogenetic analysis of the VP7 genes showed that our G12 P[6] strains belonged to lineage III and formed an independent cluster; these strains shared 100% nucleotide identity. A strain from China was close to this cluster and shared 99.6% identity with Sabahan strains (Fig 4). The only G8P[8] strain from Sabah formed a cluster with strains from Thailand, Japan, Vietnam and Czech Republic (Fig 5). These strains shared 99.5–99.7% nucleotide identity among themselves.

**Fig 6:**
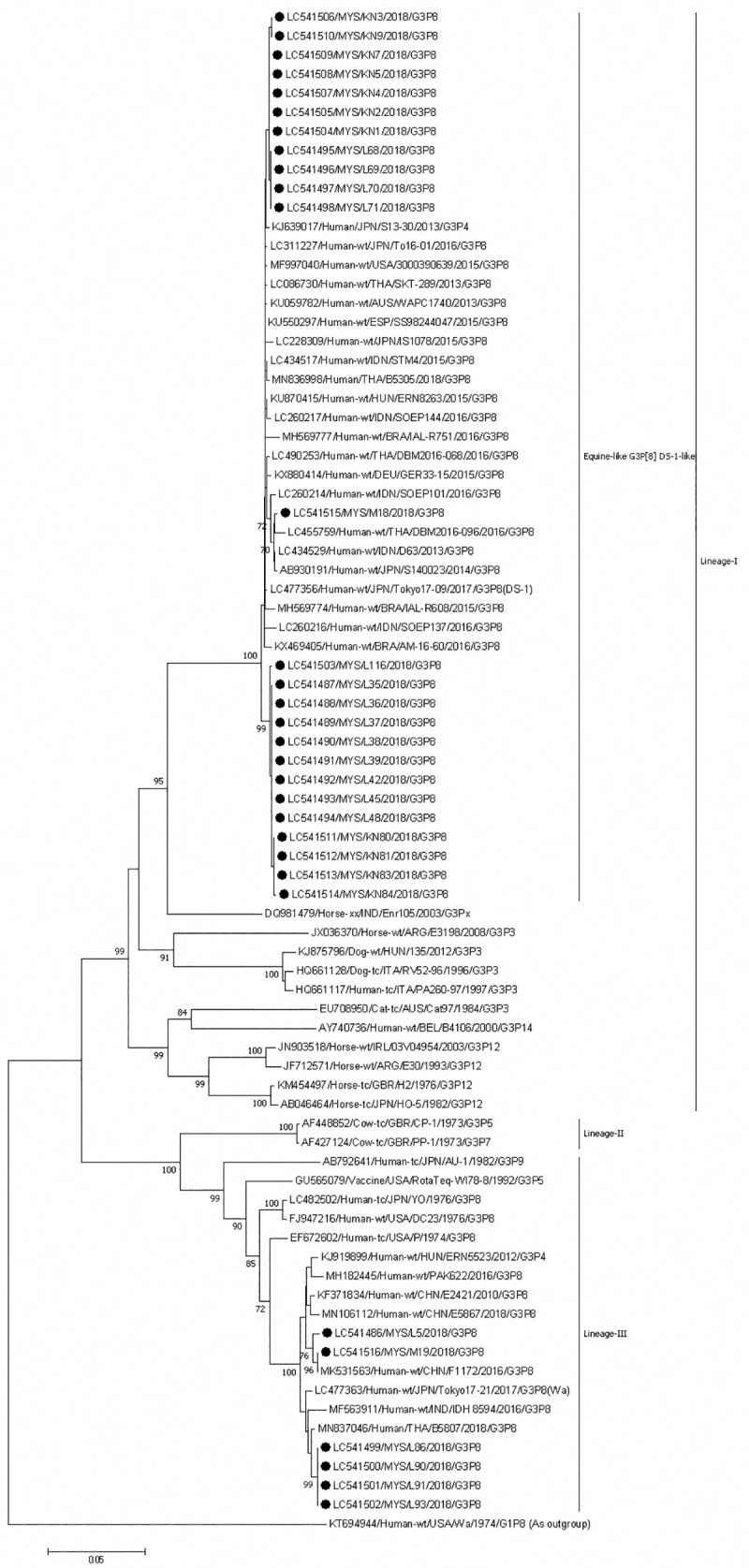
Phylogenetic tree constructed with the nucleotide sequences of the VP7 gene of G3 strains. Human rotavirus Wa-40-AG (G1) was used as an outgroup. The number adjacent to the node represents the bootstrap value and values lower than 70% have not been indicated. Scale bar shows genetic distance expressed as nucleotide substitutions per site. The strains identified in this study are marked with a filled circle. Strains from Sabah belong to lineages I and III. The nucleotide sequences of our strains have been submitted to the databases of the DNA DataBank of Japan, the European Molecular Biology Laboratory, and GenBank. The accession numbers are shown at the beginning of each strain.

The G3 strains from Sabah are divided into lineages I and III. Lineage I includes equine-like G3 strains (n = 25, 59.5%) of human origin and from horses and other animals such as dogs and cats. Lineage III contains only human G3 strains (Fig 6). The equine-like G3 strains from Sabah were divided into three clusters. In one cluster, although the Sabah strains grouped together, they were closely related to strains from Japan, Hungary, Thailand, Spain, Australia, Brazil, USA and Indonesia. Sabahan strains had 99.7–100% and 99–99.8% nucleotide identity among themselves and with strains from those countries, respectively. The other cluster contained one strain from Sabah and others from Indonesia, Thailand and Japan. These strains shared 99.2–99.6% nucleotide identity among themselves. The third cluster contained strains from Sabah only and shared 99.5–100% nucleotide identity among themselves. Strains in lineage III were divided into two clusters; one contained only strains from Sabah (99.8–100% nucleotide identity), the other contained strains from China (99.4–100% nucleotide identity).

**Fig 7:**
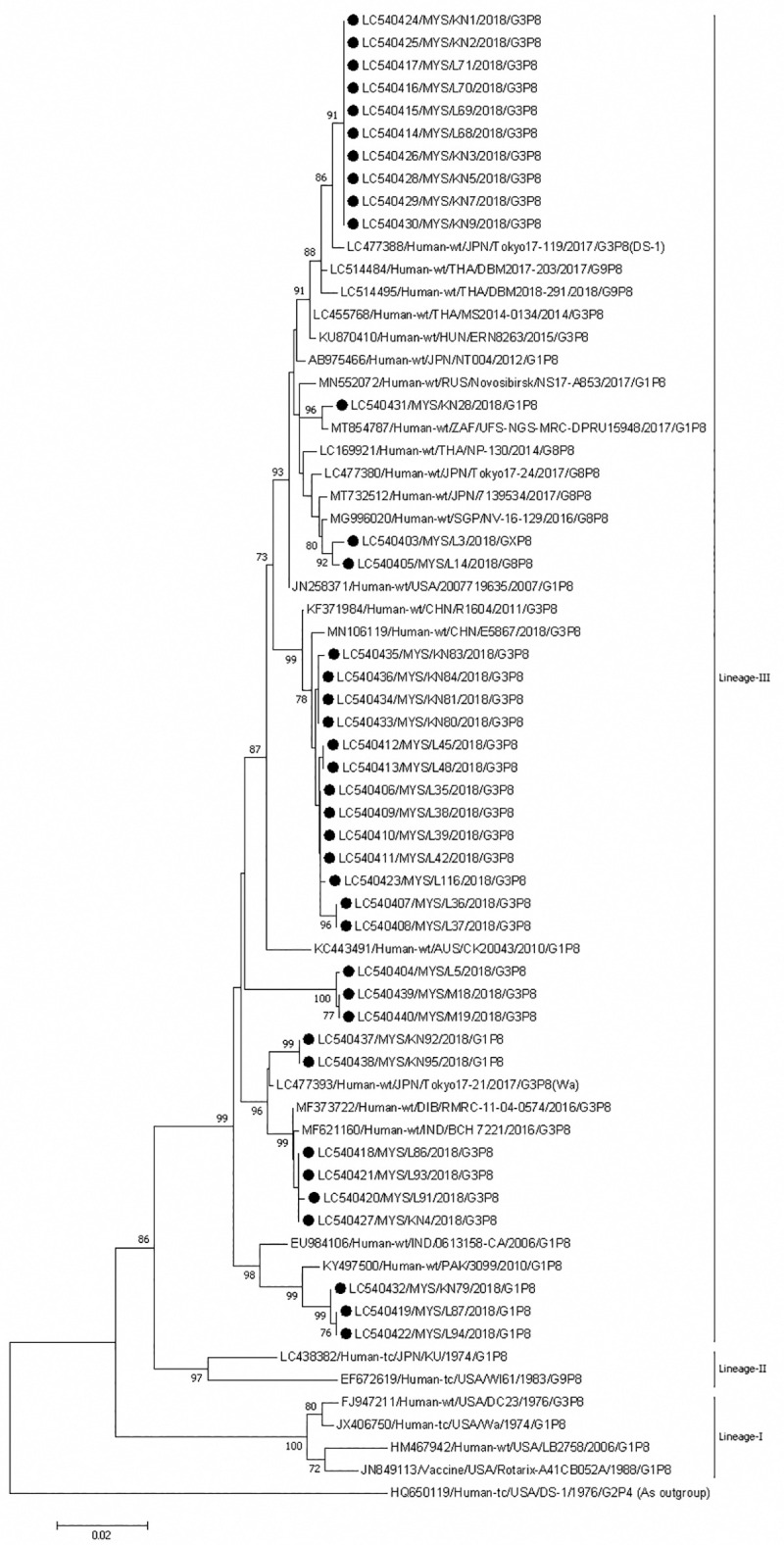
Phylogenetic tree constructed with the nucleotide sequences of the VP4 gene of P[8] strains. Human rotavirus DS-1 (P[4]) was used as an outgroup. The number adjacent to the node represents the bootstrap value and values lower than 70% have not been indicated. Scale bar shows genetic distance expressed as nucleotide substitutions per site. The strains identified in this study are marked with a filled circle. All strains from Sabah belong to lineage III. The nucleotide sequences of our strains have been submitted to the databases of the DNA DataBank of Japan, the European Molecular Biology Laboratory, and GenBank. The accession numbers are shown at the beginning of each strain.

The VP4 gene of the P[8] rotaviruses all belonged to lineage III (Fig 7). There were two clusters containing only equine-like G3 rotaviruses from Sabah. The strains in these two clusters also formed clusters in the VP7 phylogenetic tree. The first cluster is closely related to strains from Japan, Thailand and Hungary, as in the VP7 tree. The VP4 gene of Sabahan strains shared 100% and 99.5% nucleotide identities among themselves and with a strain from Japan. The second cluster was very closely related to strains from China but was an independent cluster in the VP7 tree. The VP4 gene of Sabahan strains shared 99.4–100% and 99.1–99.6% nucleotide identities among themselves and with a strain from China. An independent cluster was formed by three G3 that also contained an equine-like G3 (strain MS18). These strains shared 99.9–100% nucleotide identity among themselves. Another cluster contained three G3 and one equine-like G3 (KN4) and strains from India. The VP4 gene of Sabahan strains shared 99.9–100% and 99.6–99.9% nucleotide identities among themselves and with Indian strains. The equine-like G3 formed a cluster with these Indian strains in the VP7 phylogenetic tree. The VP4 gene of the P[8] strains of G1 formed three clusters. In one cluster, a G1P[8] rotavirus from Sabah clustered with a South African G1P[8] rotavirus. These strains shared 99.6% nucleotide identity. This South African strain was the same one that this Sabahan strain clustered in VP7 phylogenetic tree. Another cluster was formed by two G1 strains from Sabah with a 100% nucleotide identity and was very close to a G3P[8] strain from Japan, indicating a possible common origin of the VP4 gene of P[8] for these G1 and G3 rotaviruses. Also, another cluster was formed by three G1P[8] rotaviruses from Sabah, which was very close to an Indian and a Pakistani G1P[8] rotavirus. The VP4 gene of Sabahan strains shared 99.9–100% and 98.1–98.9% nucleotide identities among themselves and with a strain from Pakistan. The VP7 phylogenetic tree of these G1 was also close to a different Pakistan strain of G1P[8]. Two G8P[8] and GxP[8] strains from Sabah formed an independent cluster but were very close to a G8P[8] strain from Singapore; however, in the VP7 phylogenetic tree, this Sabahan G8 strain clustered with strains from Japan, Vietnam and Czech Republic. The VP4 gene of Sabahan strains shared 99.6% and 99.4–99.5% nucleotide identities among themselves and with a strain from Singapore. The G8P[8] and GxP[8] strains were very similar to each other, indicating the possibility of another G8 strain.

**Fig 8:**
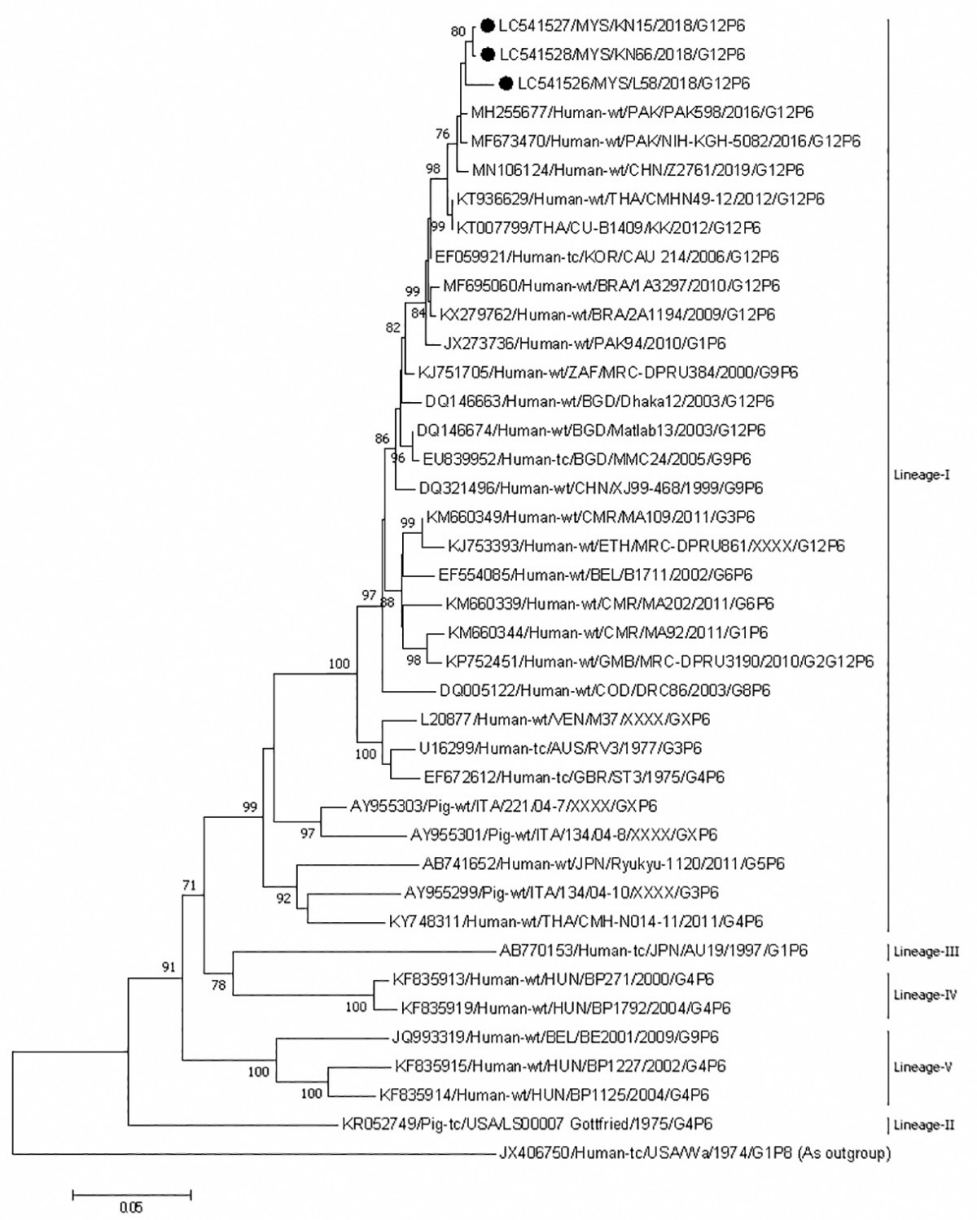
Phylogenetic tree constructed with the nucleotide sequences of the VP4 gene of P[6] strains. Human rotavirus Wa (P[8]) was used as an outgroup. The number adjacent to the node represents the bootstrap value and values lower than 70% have not been indicated. Scale bar shows genetic distance expressed as nucleotide substitutions per site. The strains identified in this study are marked with a filled circle. All strains from Sabah belong to lineage I. The nucleotide sequences of our strains have been submitted to the databases of the DNA DataBank of Japan, the European Molecular Biology Laboratory, and GenBank. The accession numbers are shown at the beginning of each strain.

The P[6] belonging to lineage I of our G12P[6] strains was close to that of human G12P[6] strains from Pakistan, and China (Fig 8). The Sabahan strains shared 98.5–99.8% and 97.8–99.3% nucleotide identities among themselves and with Pakistani and Chinese strains, respectively. The Chinese strain was the same that clustered together with Sabahan strains in the VP7 phylogenetic tree.

**Fig 9:**
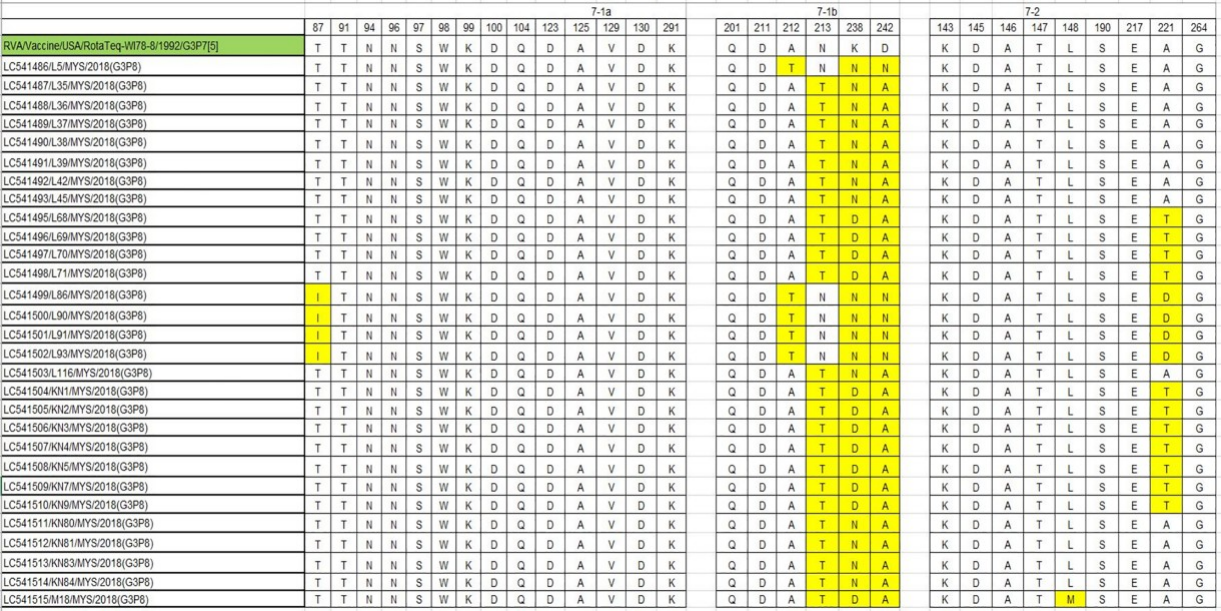
Comparison of the antigenic residues of VP7 present in genotype G3 strains of RotaTeq and the strains circulating in Sabah, Malaysia. The respective antigenic epitopes are shown above the residue numbers. The amino acid residues in the Sabahan strains that differed from those in the vaccine strains are highlighted in yellow.

In the present study, we compared the VP7 and VP4 (VP8*) antigenic epitopes of the Sabahan G3 and equine-like G3 strains with those of the RotaTeq (WI78-8) vaccine strain. Within the 29 amino acid residues comprising the VP7 antigenic epitopes, seven differences were found in our strains (Fig 9). In equine-like G3 strains only, one substitution (T87I) occurred in the 7-1a region. Within the 7-1b epitope of equine-like G3 and G3 strains, four substitutions (A212T, N213T, K238D/K238N, D242A) and three substitutions (A212T, K238N, D242N) were detected, respectively. Within the 7–2 epitope of equine-like G3 and G3 strains two (A221T and L148M) and one substitution (A221D) were detected, respectively.

**Fig 10:**
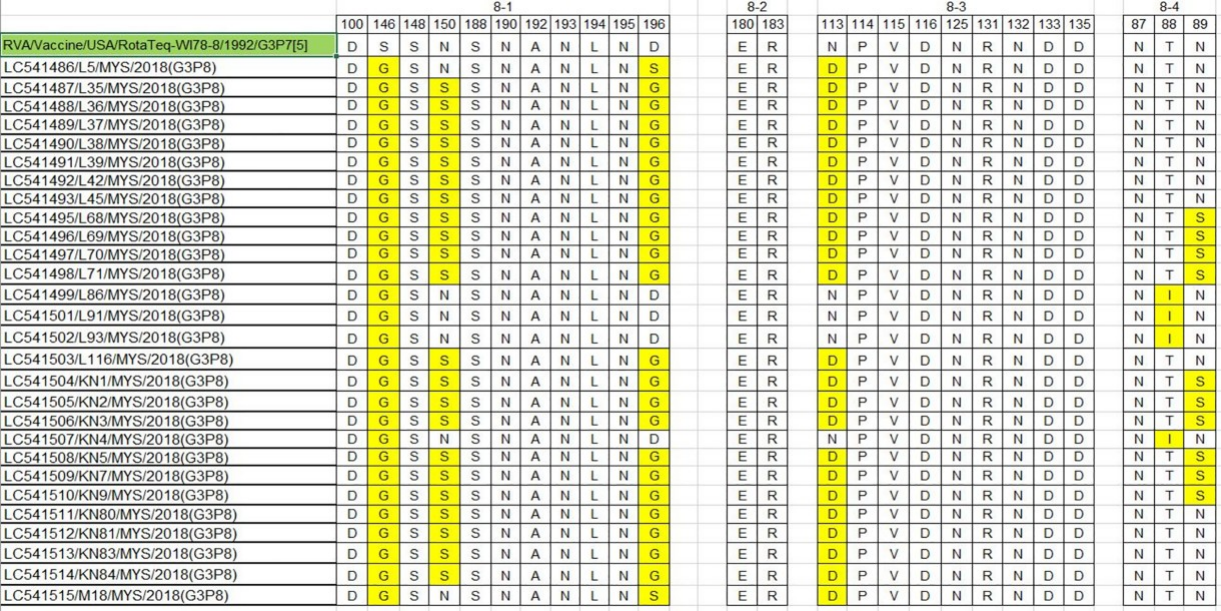
Comparison of the antigenic residues of VP4 present in genotype G3 strains of RotaTeq and the strains circulating in Sabah, Malaysia. The respective antigenic epitopes are shown above the residue numbers. The amino acid residues in the Sabahan strains that differed from those in the vaccine strains are highlighted in yellow.

Of the 25 amino acid residues comprising the VP4 antigenic epitopes, six differences were found in our strains (Fig 10). Within the 8–1 epitope of G3 and equine-like G3 strains, two substitutions (S146G, D196S) and three substitutions (S146G, N150S, D196S/D196G) were detected, respectively. No substitution was detected in the 8–2 epitope. Within the 8–3 epitope, one substitution (N113D) was detected in the equine-like G3 and G3 strains. Within the 8–4 epitope of G3 and the equine-like G3 strains, one (T88I) and two substitutions (T88I and N89S) were detected, respectively.

## Discussion

Rotavirus genotypes G1–G4, G9 and G12 in combination with P[8], P[6] and P[4] are responsible for more than 90% of rotavirus diarrhea cases worldwide [18]. Sporadic incidence of infection with rare genotypes like G5, G6, G8, G10, G11, P[1], P[3], P[9], P[10], P[19], P[14] and P[25] have been detected [11,18,27,32–36]. In general, the G and P distribution patterns of rotavirus fluctuates place to place and year to year [37]. Studies done in Malaysia from 1977 to 2010 showed that G1P[8] and G4P[8] were codominant followed by G9P[8], G2P[4], and G3P[8] [22]. Although G3 was never a predominant genotype in those studies they did not include Sabah. In the present study, we found that equine-like G3P[8] is the predominant (59.5%) genotype circulating in Sabah.

Phylogenetic analysis clearly shows that several strains of both human G3P[8] and equine-like G3P[8] are circulating among children in Sabah, indicating the presence of strains found only in Sabah and other strains that have possibly invaded Sabah from different sources, after which local spread occurred and they spread across the state. During the period of our study (2018–2019) human G3P[8] was the dominant genotype in Bangladesh; those strains were phylogenetically related to the Indian strains circulating in 2015–2017 [38,39]. In Pakistan and Myanmar, human G3P[8] was also predominant during 2016 and 2017, respectively [6,40]. However, these strains were not related to those from Sabah; rather, the latter were ether independent or related to Chinese strains.

Sabahan equine-like G3P[8] rotaviruses exhibited short electropherotypes similar to those of the Thai equine-like G3P[8] that was dominant during 2015 and 2016 [37]. However, only one of our strains was clustered with this strain in the phylogenetic tree. Our other equine-like G3P[8] belonged to independent clusters, indicating that ancestors of these clusters possibly entered Sabah in the past and evolved. Equine-like G3P[8] with short electropherotypes emerged in Australia and Thailand in 2013, in Spain and Hungary in 2015, and in Brazil, Indonesia and Japan in 2016 [37]. Whole-genome sequence analyses revealed that these equine-like G3P[8] with short electropherotypes had a DS-1-like backbone; i.e., a G3P[8]-I2-R2-C2-M2-A2-N1/2-T2-E2-H2 constellation [37]. In neighboring Indonesia’s Central Java and Yogyakarta areas, equine-like G3P[8] with G3P[8]-I2-R2-C2-M2-A2-N1/2-T2-E2-H2 constellation has become dominant and has been identified in samples from 2014 and 2015 [41]. A study from Surabaya, Indonesia, identified two distinct equine-like G3P[8] and G3P[6] strains with short electropherotypes circulating in 2015–2016 [42]. It is noteworthy that the Thai equine-like strains possessed distinct NSP4 genes: one bovine-like and the other human-like [37]. As we are not sure which of these our strains belong to, in future, the whole genome sequence analysis of equine-like G3 strains can be performed to clarify about the reassortment events.

Although equine-like G3P[8] has been detected in several countries, the detection rate varies. Few equine-like G3P[8] strains have been detected in Germany, Hungary, Japan and the USA [37]. In Australia and Spain, the detection rate was moderate, between 14.4 and 37.4%. Equine-like G3[P8] was predominant in Brazil [42,43], Indonesia [44] and Thailand during the 2016–2017 seasons [37]. In Australia, the dominance of equine-like G3P[8] is attributed to high Rotarix vaccine coverage-related vaccine-induced selective pressure [45]. The dominance of equine-like G3P[8] in Thailand and Hungary is also attributed to vaccine-induced selective pressure, although Rotarix is only available on the private market and has lower national coverage in these two countries [45]. Furthermore, the equine-like G3P[8] dominance in Spain is also attributed to vaccine-induced selective pressure, although RotaTeq has been primarily used there [45]. In Malaysia, the national coverage of rotavirus vaccine usage is unknown [46]. Because no trial was conducted in Malaysia, it is not clear how effective these vaccines will be in the local context.

To shed light on vaccine effectiveness we compared the antigenic epitopes of our G3 and equine-like G3 strains with that of the RotaTeq G3 vaccine strain and identified a number of substitutions. Although the number of substitutions in the 7-1a epitope was small, several substitutions were found on epitope 7-1b (aa208-223), which might lead to a ten-fold increase in resistance toward the binding of the neutralizing antibody [47]. The substitution K238N in this epitope is also known to be associated with a potential *N*-linked glycosylation site [48], which would prevent neutralizing antibody activity [49]. Substitution (K238N) was previously identified in Belgian [48], Iranian [50], Russian [51] and Tunisian [52] strains. Amino acid changes at residues 100, 146, 148, 150, 188, 190, 194, 180, 183, 114, 116, 133, 135, 87, 88, 89 have been shown to cause escape from neutralization with monoclonal antibodies [53]. In this study, the VP4 of Sabahan G3 and equine-like G3 strains that underwent substitutions at aa146, 150, 88, 89 have the potential to escape neutralization by vaccine-induced antibodies.

As in many other countries, in Malaysia, the prevalence of rotavirus infection varies regionally. Previous studies showed that the prevalence of rotavirus infection is the highest in Penang (54%), followed by Kuching, Sarawak (46%), and Kuala Lumpur (22–46%), then Johor Bahru, Johor (18%), with the lowest in Kota Kinabalu (16%) [26]. This previous result from Kota Kinabalu is consistent with our results. Although the age prevalence of rotavirus infection varies between developed and developing regions, it generally occurs among children 4- 23months of age [27,54–56]. We found most of the rotavirus infections in children 6–23 months old (Fig 1), with a peak at 12–17 months old. These findings are similar to those of a study in Bangladesh, where rotavirus mainly affected children 3-23-month old children and peaked at 6-11-month-old [11].

The number of rotavirus infections was lowest in patients <3 months of age, presumably because of the presence of maternal antibodies from breastfeeding. As found in our study, male patients have been reported as more likely to be affected with rotavirus acute gastroenteritis [57–59]. Similar to the findings in the countries of the Indian subcontinent [11,34], the G12 detected in Sabah were in combination with P[6]. However, our G12 strains formed an independent subcluster with G12P[6] strains from China. The P[6] genotype is mainly distributed in pigs [60–62]. Studies have shown that uncommon rotaviruses with P[6] genotypes emerged in human populations as a result of genetic reassortment between human and porcine rotaviruses [36]. The phylogenetic analysis also showed that the Sabahan P[6] was similar to human P[6] strains rather than porcine P[6] strains, although human P[6] was originally derived from porcine P[6] strains.

Genotype G9P[8] is the fifth most predominant G genotype globally, and is one of the common circulating genotypes in developing countries [63,64]. In the present study, no G9 was identified; however, the G9 genotype was identified previously as the predominant genotype in Johor, West Malaysia [23].

Another significant finding in this study is the identification of a G8P[8] strain. The unexpected emergence of a rotavirus G8 strain for the first time in Malaysia has raised the epidemiologic significance of this strain. G8 was initially identified in neighboring Indonesia [65] and later in other countries [66–69] but has never been reported in Malaysia. In Africa, childhood infections caused by G8 strains are more common, accounting for 5–20% of the strains, and are frequently associated with P[6], P[4], or P[8] [70]. The identification of closely related G8P[8] strains in Vietnam, Japan and Thailand indicates that they are spreading in Asia [69,71,72], suggesting a common origin. According to VP7 sequence analysis, the G8 strains from Asia are related to animal strains and more distantly to G8 strains from Africa, where historically this genotype has been more commonly detected [69]. In fact, whole-genome analysis of a representative G8 strain obtained during the 2014 outbreak in Japan indicated that it emerged from the reassortment of human, sheep and bovine rotavirus genome segments [73]. It has been suggested that high vaccine coverage played a significant role in the recent emergence of G8 strains in Australia [45].

The detection of 11 different electropherotypes among 34 electropherotyped samples in our study might indicate the substantial diversity of rotaviruses circulating in Sabah compared with Turkey (5/38) [27], Sri Lanka (18/74) [35], Hong Kong (35/432) [74], Bhutan (10/38) [34] and Bangladesh (15/88) [11]. While the reason for the high diversity of rotavirus in Sabah is unknown, it indicates high rates of interaction among different strains that might be responsible for reassortant strains. Support for this speculation also comes from the phylogenetic analyses of the VP7 and VP4 genes of different strains. Some of the VP7 and VP4 components arose from the same strains, while arose from different strains, indicating the reassortment of genes. The factors behind the high diversity of strains and the unusual genotype distributions found in Sabah are complex.

There are large number of undocumented immigrants from neighboring Indonesia and the Philippines. These movements of people might contribute to the generation of diversity in the Sabahan rotavirus gene pool. In addition, 32 different ethnic groups with their different ways of life live in Sabah. Several of these, particularly in the rural areas, have close contact with animals, which creates opportunities for species jumps. Continuous surveillance of circulating rotavirus strains in Sabah is therefore important not only to monitor the pre- and post-vaccination genotype distribution, but also to monitor the emergence of new reassortant strains.

## Conclusions

In Sabah, rotavirus mainly affected children under two years of age. The predominant rotavirus circulating among the children in this study was the equine-like G3P[8] with a short electropherotype. Other genotypes circulating among the children of Sabah were G3P[8], G1P[8], G12P[6] and G8P[8]. The circulating genotypes were phylogenetically diverse and related to strains from different countries. A number of electropherotypes were also observed among Sabahan rotavirus strains. These results indicate that considerable genetic exchange is occurring in strains from Sabah. Compared with the RotaTeq vaccine strain, the antigenic epitopes present on VP7 and VP4 of Sabahan G3 and equine-like G3 differed considerably. Continuous surveillance of rotavirus strains is necessary to monitor circulating rotavirus genotypes before and after the introduction of rotavirus vaccine in Sabah.

## Supporting information

S1 Fig Map of Malaysia showing 13 states. The map was constructed using QGIS 3.18.2 software. The source file was downloaded from Natural Earth website (https://www.naturalearthdata.com/downloads/10m-cultural-vectors/).
(TIF)
