# Supplementary figures and images for: Emergence of equine-like G3 strains as the dominant rotavirus among children under five with diarrhea in Sabah, Malaysia during 2018–2019
Source: PLoS One. 2021 Jul 28;16(7):e0254784. doi: 10.1371/journal.pone.0254784 (PMC8318246; doi:10.1371/journal.pone.0254784)

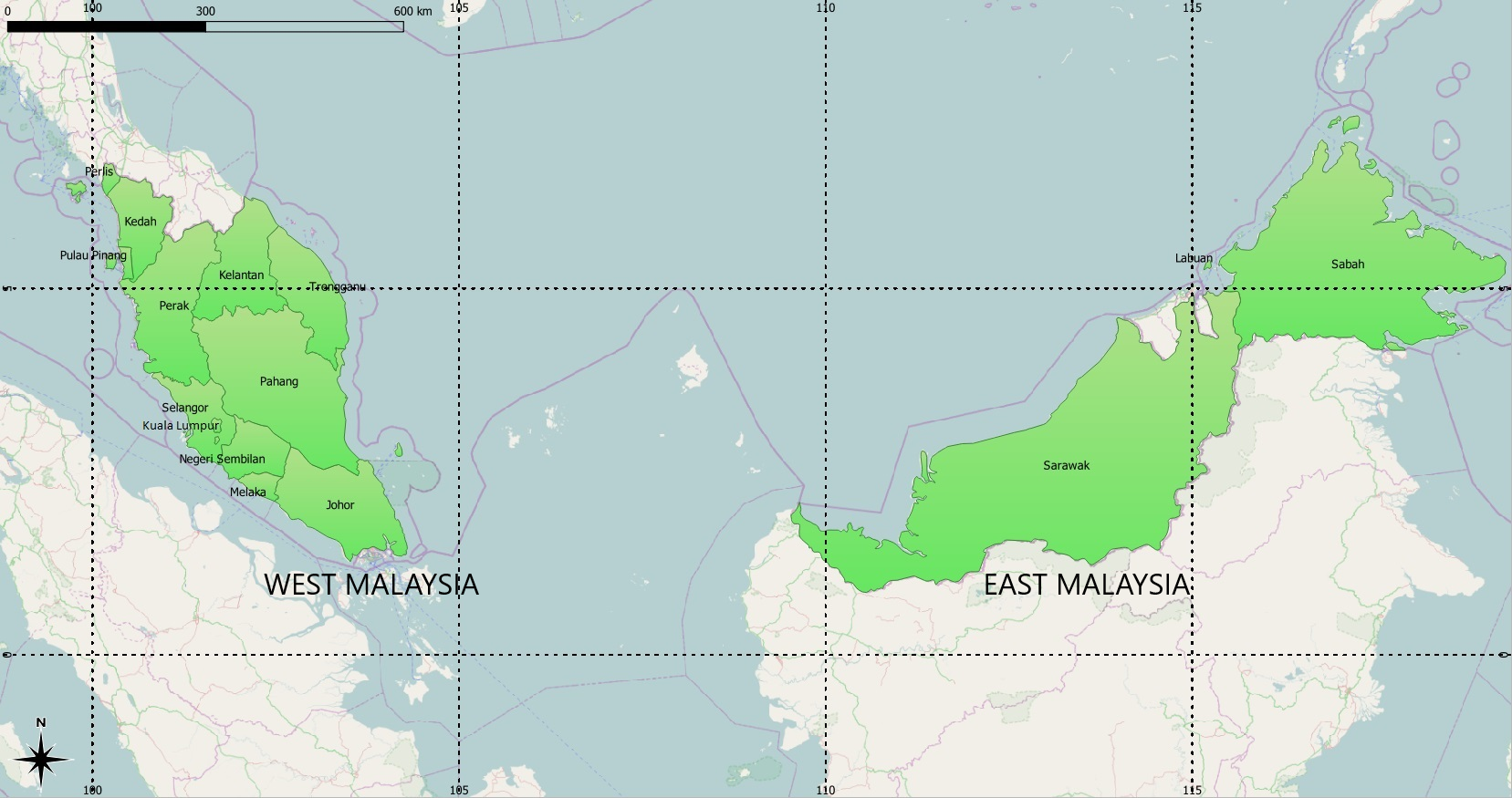

Supplement: S1 Fig — The map was constructed using QGIS 3.18.2 software. The source file was downloaded from Natural Earth website (https://www.naturalearthdata.com/downloads/10m-cultural-vectors/). (TIF) [file pone.0254784.s001.tif]
